# A Case of Euglycemic Diabetic Ketoacidosis: SGLT2 Inhibitor Complication Triggering Acute Coronary Syndrome and Cardiac Arrest Post Coronary Artery Bypass Grafting (CABG)

**DOI:** 10.7759/cureus.90044

**Published:** 2025-08-13

**Authors:** Freideriki Nteka, Philippos Alexiou, Christos Ballas

**Affiliations:** 1 Cardiac Surgery, Univeristy General Hospital of Ioannina, Ioannina, GRC; 2 Medicine, Aristotle University of Thessaloniki, Thessaloniki, GRC; 3 Cardiac Surgery, University General Hospital of Ioannina, Ioannina, GRC

**Keywords:** cardiac arrest, coronary artery bypass grafting (cabg), coronary artery bypass grafting(cabg), edka, euglycemic diabetoketoacidosis, sglt2 inhibitors, sodium-glucose cotransporter-2 (sglt-2) inhibitors

## Abstract

Sodium-glucose cotransporter-2 inhibitors (SGLT2i) are widely used in diabetes mellitus (DM) and cardiovascular disease for their glycemic and cardioprotective benefits. However, they carry an uncommon risk of euglycemic diabetic ketoacidosis (EDKA), a form of DKA with near-normal blood glucose. This complication is especially perilous in perioperative settings, and the incidence and outcomes of EDKA in cardiovascular patients have not been robustly quantified. Hence, we present a case of an elderly diabetic patient who developed EDKA on postoperative day (POD) 5 after elective coronary artery bypass grafting (CABG), which precipitated an acute coronary syndrome (ACS) and cardiac arrest.

A 79-year-old male with insulin-dependent type 2 DM (on SGLT2i therapy, empagliflozin 10 mg daily) underwent uncomplicated CABG for multi-vessel coronary disease. His SGLT2i therapy was continued until the day of surgery (no preoperative washout). On POD 5, he developed high anion gap metabolic acidosis with normal glycemia (pH=7.1, pCO₂=19 mmHg, bicarbonate (HCO₃⁻)=9 mEq/L, anion gap 26.4 mEq/L). Other causes of high-anion-gap metabolic acidosis were excluded (serum lactate 1.8 mmol/L, afebrile with normal WBC, stable renal and liver function). These findings confirmed EDKA. Despite prompt insulin and fluid therapy, the patient acutely developed chest pain and hemodynamic collapse. Emergent angiography revealed a critical stenosis of the left internal mammary artery (LIMA)-to-left anterior descending (LAD) bypass graft, confirming an ACS due to metabolic stress from EDKA rather than surgical technical failure or perioperative graft occlusion. Restoration of blood flow through the LIMA-LAD graft was achieved after deploying a coronary stent across the stenosis. The patient stabilized and made a gradual recovery.

This case illustrates that EDKA can impose severe metabolic stress on the heart, effectively acting as a “cardiac stress test” that triggers myocardial ischemia and arrhythmias. Current guidelines recommend discontinuing SGLT2i at least three days before major surgery to mitigate this risk. Clinicians must maintain a high index of suspicion for EDKA in postoperative patients on SGLT2i therapy after CABG surgery, as early recognition and treatment are critical to preventing catastrophic outcomes. Furthermore, the case highlights the importance of multidisciplinary management in cardiovascular patients perioperatively to mitigate the risk of EDKA and its life-threatening cardiac sequelae.

## Introduction

Euglycemic diabetic ketoacidosis (EDKA) is a rare but life-threatening complication of diabetes mellitus (DM) characterized by the triad of high anion gap metabolic acidosis, ketosis, and normal or mildly elevated blood glucose [1]. It accounts for approximately 2.6% to 3.2% of all DKA cases. Unlike classic DKA, EDKA can be easily missed because patients do not present with extreme hyperglycemia, and the usual osmotic symptoms may be attenuated [2]. Various stressors can precipitate EDKA, including fasting, surgical stress, infection, or acute illness, all of which may occur in the perioperative period. Notably, the newer class of oral antihyperglycemics, sodium-glucose cotransporter-2 inhibitors (SGLT2i), has been implicated in precipitating EDKA in both type 1 and type 2 DM [3]. SGLT2i (such as empagliflozin, dapagliflozin, and canagliflozin) reduce renal glucose reabsorption and have proven cardiovascular benefits, whereas by lowering insulin levels and raising glucagon levels, they create a metabolic milieu prone to ketosis [4].

Patients on SGLT2i who undergo major surgery are at particular risk for EDKA due to perioperative fasting and stress hormone surges. Post-cardiac surgery patients often have multiple potential causes of metabolic acidosis (e.g., hypoperfusion, lactate from cardiopulmonary bypass, etc.), which can mask the diagnosis of EDKA. Thus, EDKA in this setting is a potentially catastrophic complication, requiring a high index of suspicion for timely diagnosis [5]. In fact, EDKA not only causes metabolic derangements but can also have cardiovascular consequences. Severe acidosis, dehydration, and electrolyte shifts in EDKA put significant stress on the myocardium, which may provoke arrhythmias or demand ischemia. Recent guidelines recommend discontinuing SGLT2i at least three days before elective procedures (four days for ertugliflozin) to reduce the risk of perioperative ketoacidosis. Nevertheless, cases continue to emerge, most commonly between postoperative days (PODs) 3 and 7, underscoring a vulnerable risk window in the early postoperative period [6].

There is growing awareness that EDKA can sometimes act as a trigger for cardiac events, effectively unmasking latent coronary insufficiency. Ketosis and systemic acidosis promote a pro-inflammatory and pro-thrombotic state by activating endothelial cells and platelets, while acidemia impairs nitric oxide-mediated vasodilation, favoring coronary vasoconstriction. Concurrently, the counter-regulatory surge in catecholamines during DKA elevates heart rate and myocardial oxygen demand, precipitating a supply-demand mismatch in patients with underlying coronary disease. In addition, the shift of myocardial metabolism toward free fatty acids and ketone bodies under low-insulin conditions reduces cardiac efficiency in ischemic myocardium. These factors, taken together, create a perfect storm in which EDKA can unmask or exacerbate coronary insufficiency. Recent case reports have described instances of acute coronary syndrome (ACS) and even cardiac arrest temporally linked to SGLT2i-associated EDKA. It has been estimated that the overall perioperative EDKA incidence is approximately 0.1% to 0.3% among SGLT2i users, but data specific to cardiac surgery are scarce. [7,8]. Herein, we present a case report that exemplifies this perilous sequence, occurring after coronary artery bypass grafting (CABG) surgery, and we discuss the pathophysiological link between EDKA and cardiac ischemia, as well as preventive strategies for patients on SGLT2i.

## Case presentation

A 79-year-old male patient with a long-standing history of type 2 DM (15 years) treated with basal-bolus insulin and an SGLT2i (empagliflozin) was admitted for elective CABG. His DM had been suboptimally controlled (HbA1c = 8.5%), and he had known coronary artery disease with multivessel stenoses on angiography. Other history included hypertension and dyslipidemia, but no prior DKA episodes. The SGLT2i had been continued until the time of surgery. The patient provided informed consent for both the procedure and subsequent publication of his case.

The patient underwent elective CABG, including the following three grafts: left internal mammary artery (LIMA) graft to the left anterior descending artery (LAD), and saphenous vein grafts to the obtuse marginal and right coronary artery. The surgery was uncomplicated, and intraoperative hemodynamics were stable. He was transferred to the cardiac intensive care unit (ICU) postoperatively, where he was initially managed on ventilator support and an insulin infusion per protocol for tight glycemic control. The patient was extubated on POD 1 and transitioned to SGLT2i on POD 3. The immediate postoperative course (PODs 1-4) was uneventful.

On POD 5, the patient began to experience malaise, mild nausea, and tachypnea. Physical examination revealed deep respirations and dry mucous membranes; the patient was alert (Glasgow Coma Scale = 10/15), and vital signs showed borderline hypotension (blood pressure (BP) = 100/60 mmHg), sinus tachycardia (heart rate (HR) = 130 beats per minute (bpm)), and capillary blood glucose was 156 mg/dL. Arterial blood gas analysis, however, revealed a high anion gap metabolic acidosis: pH 7.1, pCO₂ 19 mmHg, bicarbonate (HCO₃⁻) 9 mEq/L, with an anion gap of 26.4 mEq/L. Serum lactate was only mildly elevated at 1.8 mmol/L, and troponin I was decreasing after the CABG surgery (Table 1). Given the unexpectedly severe acidosis out of proportion to lactate and the relatively elevated glycemia, EDKA was suspected. Blood tests confirmed ketosis: serum β-hydroxybutyrate was 5.5 mmol/L, and urine analysis was strongly positive for ketones. Blood cultures were negative, and there were no signs of infection, normal WBC, and no fever. The working diagnosis was EDKA precipitated by SGLT2i use in the setting of recent surgery. Prompt treatment was initiated with intravenous fluids (1 L of crystalloid bolus) and an insulin infusion (0.1 U/kg/h) alongside dextrose 5% supplementation to prevent hypoglycemia. Electrolytes were closely monitored and corrected. Over the next few hours, the patient’s metabolic parameters began to improve, arterial pH rose to 7.3, and the anion gap trended down to 14 mEq/L.

**Table 1:** Blood Laboratory Tests on Postoperative Day 5

Laboratory Test	Value	Reference Range
White Blood Cells	10.45 X 10^3^ /μL	4 – 11 X 10^3^ /μL
Neutrophils	56 %	40 – 75 %
Lymphocytes	34.2 %	20 – 45 %
Monocytes	7.5 %	2 – 10 %
Eosinophils	1.9 %	1 – 6 %
Basophils	0.4 %	0.2 – 1 %
Red Blood Cells	3.07 X 10^6^ /μL	3.8 – 6 X 10^6^ /μL
Hemoglobin	8.6 g/dL	11.8 – 17.8 g/dL
Hematocrit	27 %	36 – 52 %
Mean Corpuscular Volume	87.9 fL	80 – 96 fL
Mean Corpuscular Hemoglobin	27 pg	26 – 32 pg
Mean Corpuscular Hemoglobin Concentration	31.9 pg/dL	32 – 36 pg/dL
Platelets	233 X 10^3 ^/μL	140 – 450 X 10^3 ^/μL
INR	1	1 – 1.3
Activated Partial Thromboplastin Time	28.9 sec	26 – 36 sec
Fasting Blood Sugar	133 mg/dL	70 – 115 mg/dL
HbA1c	8.5 %	0 – 6 %
Urea	95 mg/dL	0 – 50 mg/dL
Creatinine	1.24 md/dL	0.8 – 1.4 mg/dL
Potassium	3.1 mmol/dL	3.5 – 5.1 mmol/dL
Sodium	150 mmol/dL	136 – 146 mmol/dL
Magnesium	2.7 mEq/L	1.3 – 2.1 mEq/L
Calcium	8 mg/dL	8.2 – 10.5 mg/dL
Total Proteins	4.9 g/dL	6.2 – 8.4 g/dL
Albumin	3.1 g/dL	3.5 – 5.1 g/dL
Total Bilirubin	1.3 mg/dL	0.1 – 1.3 mg/dL
Aspartate Aminotransferase	34 IU/L	5 – 40 IU/L
Alanine Aminotransferase	23 IU/L	5 – 40 IU/L
Gamma-Glutamyl Transferase	23 IU/L	8 – 45 IU/L
Alkaline Phosphatase	82 IU/L	35 – 125 IU/L
Low-density lipoprotein	391 IU/L	120 – 230 IU/L
Creatine Phosphokinase	305 IU/L	0 – 220 IU/L
Creatine Kinase–MB Isoenzyme	30 IU/L	0 – 23 IU/L
Amylase	79 IU/L	28 – 100 IU/L
Uric acid	7.1 mg/dL	3.6 – 7.8 mg/dL
Thyroid-Stimulating Hormone	1.98 μIU/mL	0.35 – 4.94 μIU/mL
B-type Natriuretic Peptide	564 pg/mL	0 - 100 pg/mL
Total Cholesterol	248 mg/dL	120 – 220 mg/dL
High-Density Lipoprotein -Cholesterol	87 mg/dL	35 – 55 mg/dL
Triglycerides	128 mg/dL	30 – 160 mg/dL
Ferritin	1240 ng/mL	0 – 300 ng/mL
Vitamin B12	194 pg/mL	187 – 883 pg/mL
CRP	16.2 mg/dL	0 – 0.8 mg/dL
Troponin I	3473 pg/mL	mg/dL
Arterial pH	7.1	7.35 – 7.45
pO_2_	75.9 mmHg	60 – 100 mmHg
pCO_2_	19 mmHg	35 – 45 mmHg
Arterial Oxygen Saturation (sO_2_)	91.5 %	60 – 100 %
Bicarbonate (HCO_3_)	9 mmol/L	18 – 25 mmol/L
Lactate	1.8 mmol/L	0.5 – 2.2 mmol/L
Anion Gap	26.4 mEq/L	8 – 16 mEq/L
β-hydroxybutyrate	5.5 mmol/L	0 – 0.5 mmol/L

**Figure 1:**
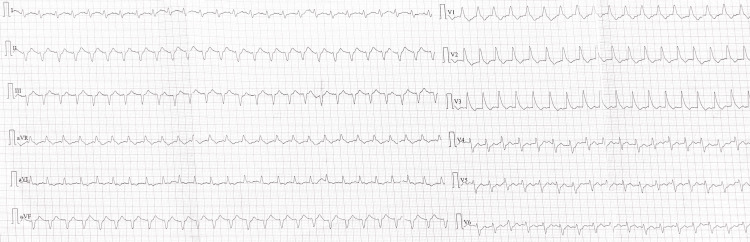
ECG on Postoperative Day 5, Presenting New Right Branch Bundle Block (RBBB), Stress-Induced Cardiac Ischemia (Heart Rate of 130 beats per minute) and ST-Segment Depressions on Leads V4-V6

Approximately six hours into EDKA treatment, the patient developed acute retrosternal chest pain, a new right branch bundle block (RBBB), and stress-induced cardiac ischemia (HR=130 bpm), and ST-segment depressions on leads V4-V6 were noted on electrocardiogram (ECG) (Figure 1), while his BP dropped to 70/40 mmHg. A repeat troponin I drawn at that time showed a continuous decrease postoperatively. Suddenly, the patient lost consciousness, and a code blue was called due to cardiac arrest; hence, cardiopulmonary resuscitation (CPR) was initiated immediately. After about two minutes of CPR and one defibrillation shock, return of spontaneous circulation was achieved. Transthoracic echocardiography (TTE) demonstrated a decline in left ventricular ejection fraction by approximately 20%, with new regional wall-motion abnormalities of the anterior wall consistent with acute ischemia. Given the history of recent CABG, an emergent decision was made to perform urgent coronary angiography to identify a graft occlusion or coronary lesion as the culprit of the arrest.

**Figure 2:**
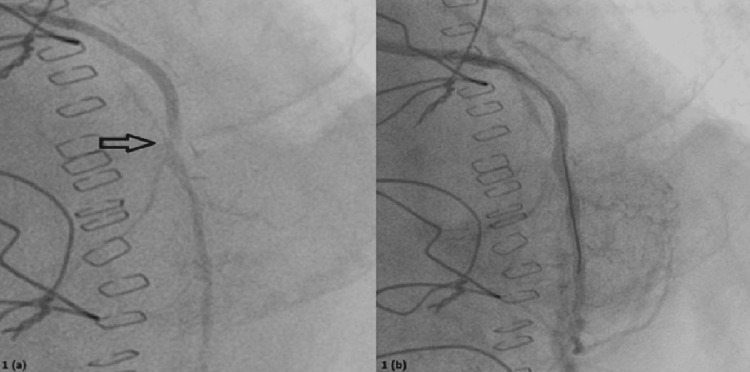
Coronary Angiography Confirmed the Critical LIMA-LAD Graft Lesion as the Culprit of Acute Coronary Syndrome. (A) Pre-intervention Angiogram Shows the Left Internal Mammary Artery (LIMA) Graft to the Left Anterior Descending Artery (LAD) With a Severe Proximal Stenosis (Arrow). There Is Markedly Reduced Flow Beyond the Stenotic Anastomosis Into the LAD Territory. (B) Post-intervention Angiogram Demonstrates Restoration of Blood Flow Through the LIMA-LAD Graft After Deploying a Coronary Stent Across the Stenosis.

On angiography, the vein grafts to the obtuse marginal and right coronary artery were patent. However, the LIMA-LAD arterial graft showed a critical stenosis just at the anastomosis with the LAD. The native LAD beyond the anastomosis was small caliber with diffuse disease. This finding indicated that the anterior wall was dependent on the compromised LIMA graft, explaining the anterior infarction. The critical graft stenosis was the precipitant of the ACS and arrest in the setting of stress. A decision was made to intervene percutaneously, and three coronary stents were deployed across the stenosis to restore the blood flow due to the tortuosity of the vessel (Figure 2). The angiographic result confirmed successful treatment of the graft stenosis and improved perfusion to the anterior wall.

The patient was managed in the cardiac ICU following the angiographic intervention. Given cardiogenic shock (cardiac index 1.8 L/min/m², pulmonary capillary wedge pressure 25 mmHg, mean arterial pressure (MAP) 60 mmHg despite high-dose vasopressors), an intra-aortic balloon pump (IABP) was inserted for mechanical circulatory support. The patient stabilized over the next 24 hours, and the EDKA steadily resolved with the continuation of insulin, crystalloid fluids, and electrolyte replacement. Anticoagulation post-percutaneous coronary intervention balanced bleeding risk from recent surgery with stent thrombosis prevention with unfractionated heparin for 24 hours and single antiplatelet therapy (clopidogrel 75 mg daily). During his stay in the cardiac ICU, the patient presented multiple episodes of atrial fibrillation (AF), which were managed with intravenous amiodarone 300 mg x 3 for three days and anticoagulant therapy (apixaban 5 mg x 2). By POD 8, the arterial blood gas analysis was unremarkable. TTE after the event showed moderate left ventricular systolic dysfunction (ejection fraction = 40% vs. 50% preoperatively), consistent with a recent infarction, but no mechanical complications. Neurologically, the patient made an excellent recovery with no deficits, given the short duration of CPR. He was extubated (for the second time) two days post arrest and mobilized gradually. Prior to discharge, the SGLT2i was permanently discontinued, and he was maintained on a basal-bolus insulin regimen with close glucose monitoring. In addition, the patient received antiplatelet and anticoagulant therapy (clopidogrel 75 mg x 1 and apixaban 5 mg x 2), considering the stented graft and postoperative atrial fibrillation, along with a high-intensity statin and an angiotensin-converting enzyme inhibitor (ACEi). The patient was discharged home on POD 12 in stable condition. At follow-up one month later, the patient was recovering well with no recurrent angina and good glycemic control.

## Discussion

This case highlights an uncommon but significant complication of SGLT2i-associated EDKA, precipitating an ACS and cardiac arrest after CABG surgery. While DKA is classically considered a consequence of acute illnesses, here the causality appears reversed; the metabolic derangement from EDKA served as the trigger for myocardial ischemia. We propose that the severe metabolic stress of EDKA placed an extreme demand on the patient’s cardiovascular system, effectively acting as a “cardiac stress test” that the recently grafted coronary circulation could not withstand. In the context of a critical LIMA-LAD graft stenosis, the surge in catecholamines and tachycardia during EDKA provoked a supply-demand mismatch or plaque instability that culminated in an anterior wall infarction and malignant arrhythmia. This concept of EDKA precipitating cardiac ischemia has been suggested in prior reports [9].

SGLT2i-associated EDKA is pathophysiologically linked to several factors that can have an adverse impact on the heart. Firstly, acidosis and ketosis reduce myocardial contractility and can cause vasodilation, potentially lowering coronary perfusion pressure [10]. Furthermore, severe acidosis sensitizes the myocardium to arrhythmias and reduces the threshold for ventricular fibrillation. The counter-regulatory surge in catecholamines and glucagon during EDKA exacerbates tachycardia and BP lability, increasing cardiac workload [11]. The SGLT2i effect of osmotic diuresis leads to intravascular volume depletion, which in a postoperative patient can worsen perfusion of vital organs, including the heart. This hypovolemia, combined with relative insulinopenia, shifts myocardial metabolism towards free fatty acids and ketone bodies, further straining the myocardium’s energy balance [12].

It is also worth noting that EDKA itself can elevate cardiac biomarkers and cause transient wall motion abnormalities even without fixed coronary lesions (pseudoinfarction patterns) [13]. In our patient, however, the angiographic finding of a critical graft stenosis provides a concrete link between the metabolic stress and a true ischemic substrate. This underscores the importance of thorough cardiac evaluation in any EDKA episode in at-risk cardiovascular patients. In scenarios like ours, simultaneous management of EDKA and prompt cardiac assessment is warranted to address both the metabolic and ischemic aspects.

Current perioperative guidelines reflect growing recognition of this risk. It is now recommended to withhold SGLT2i for at least 72 hours before elective surgery, and for 96 hours in the case of ertugliflozin, while resuming only after the patient has stable oral intake and normal acid-base status. These intervals are based on the pharmacokinetics of the drugs and observational data linking shorter washout periods to higher EDKA incidence. Given this risk window, it is prudent to implement a ketone and anion gap monitoring protocol for high-risk patients post CABG. For example, measuring blood β-hydroxybutyrate and basic metabolic panel every 12 hours for the first 72 hours after surgery can facilitate early detection of rising ketones or widening anion gaps before overt acidosis develops.

Finally, multidisciplinary perioperative planning is critical; involving endocrinology to advise on safe timing of antidiabetic medication holds, anesthesia to anticipate fasting-related catabolism, and cardiology to review graft patency and hemodynamic goals ensures that patients at risk for EDKA and cardiac complications receive coordinated care. By integrating these preventive and monitoring strategies, clinicians can mitigate the substantial risk of EDKA-triggered ACS in the perioperative setting [14].

## Conclusions

In conclusion, this case highlights the critical need for early recognition and aggressive management of EDKA in postoperative patients after CABG surgery, particularly those on SGLT2i. EDKA may present subtly but can rapidly progress to life-threatening situations, including the precipitation of acute myocardial ischemia and arrhythmias. By rigorously applying guideline-endorsed discontinuation intervals, integrating multidisciplinary coordination, and employing targeted postoperative monitoring and follow-up, clinicians can decisively reduce the risk of EDKA-triggered ACS and cardiac arrest in cardiovascular patients on SGLT2i.
